# The Effect of spinal anesthesia on sensory and motor block in cesarean cases with a diagnosis of gestational diabetes mellitus

**DOI:** 10.12669/pjms.41.10.12138

**Published:** 2025-10

**Authors:** Tuna Albayrak, Dilek Yeniay

**Affiliations:** 1 Tuna Albayrak Department of Anesthesiology and Reanimation, Giresun Training and Research Hospital, Giresun, Turkey; 2 Dilek Yeniay Department of Anesthesiology and Reanimation, Giresun Training and Research Hospital, Giresun, Turkey

**Keywords:** Cardiovascular Response, Gestational Diabetes Mellitus (GDM), Motor Block Duration, Spinal Anesthesia, Sensory Block Duration

## Abstract

**Objective:**

Gestational diabetes mellitus (GDM) poses challenges in managing anesthesia, particularly during cesarean sections where spinal anesthesia is commonly used. Physiological changes associated with GDM, such as neuropathy and cardiovascular complications, can influence the effectiveness and outcomes of anesthesia. This study aimed to compare the cardiovascular and respiratory responses, as well as the durations of sensory and motor blocks, in patients with GDM and those without undergoing cesarean sections under spinal anesthesia.

**Methodology:**

This prospective, controlled, observational study was conducted at Giresun Women’s Health and Research Hospital from April 2023 to August 2024. A total of 56 patients (28 with GDM and 28 without) undergoing elective cesarean section with spinal anesthesia were enrolled. Spinal anesthesia was administered using a standard dose of local anesthetic (12 mg of 0.5% hyperbaric bupivacaine). The recovery durations of sensory and motor blocks, the time to first postoperative analgesic requirement, and hemodynamic parameters (heart rate and blood pressure) were recorded.

**Results:**

Patients with GDM demonstrated significantly prolonged sensory and motor block durations compared to non- GDM patients (p<0.05). Furthermore, GDM patients exhibited delayed cardiovascular responses, presenting significantly higher heart rates at the 10th minute and increased mean arterial pressure (MAP) at the 30th and 45th minutes.

**Conclusion:**

This study indicates that patients with GDM experience extended block durations and altered cardiovascular responses, highlighting the necessity for tailored anesthesia protocols. Larger multicenter studies are required to validate these findings and explore the impacts of other anesthesia types, such as epidural and general anesthesia.

## INTRODUCTION

Gestational diabetes mellitus (GDM) is a condition characterized by glucose intolerance, which develops during pregnancy and can lead to various maternal and fetal complications. The prevalence of GDM has been increasing worldwide due to rising rates of obesity and advancing maternal age. As a result, it is critical to understand how GDM influences physiological responses, especially in perioperative settings, where anesthesia management plays a vital role in patient outcomes.^1^-^5^

Anesthesia for patients with GDM presents challenges, particularly because GDM is associated with several complications including autonomic dysfunction, diabetic neuropathy, and altered cardiovascular and respiratory functions. These complications can significantly affect the administration of anesthesia, especially regional anesthesia, which is commonly used during cesarean sections and other surgical procedures in pregnant women.^6^ Diabetic neuropathy, a well-documented complication of diabetes, affects nerve sensitivity and response to anesthetics, leading to prolonged sensory and motor block durations. Autonomic dysfunction can impair cardiovascular responses during surgery, necessitating careful monitoring and individualized anesthesia management.^7^

Previous studies have explored the effects of diabetes on motor and sensory block durations in peripheral nerve blocks.^3^,^4^,^8^,^9^ However, there is limited research on the impact of GDM on spinal blocks, particularly regarding the effect of GDM on cardiovascular and respiratory responses during anesthesia. Despite the growing incidence of GDM and its potential to complicate anesthetic management, studies specifically addressing spinal anesthesia outcomes in this patient group remain scarce. This gap in knowledge limits the ability of clinicians to anticipate intraoperative challenges and optimize anesthetic protocols. Understanding these physiological changes is critical for optimizing anesthetic care and ensuring the safety of GDM patients undergoing surgery.

This study aimed to compare the cardiovascular and respiratory responses, as well as the sensory and motor block durations, in patients with and without GDM undergoing regional anesthesia. By investigating these differences, we hope to provide valuable insights into the management of anesthesia in GDM patients and contribute to the growing body of knowledge on the interplay between GDM and anesthetic response. In doing so, the present study addresses a crucial gap in the literature and highlights the clinical relevance of tailoring anesthesia strategies to the specific needs of GDM patients. Furthermore, this study seeks to address the gaps in the literature regarding the influence of GDM on spinal blocks and the potential for increased risks during surgical procedures.

## METHODOLOGY

This study aimed to investigate whether there are differences in the duration of sensory and motor block recovery between GDM and non-GDM patients undergoing cesarean section under spinal anesthesia. Additionally, we examined the effect of GDM on the time to the first postoperative analgesic requirement.

This prospective, controlled, observational study was conducted at Giresun Women’s Health and Research Hospital between April 2023 and August 2024. A total of 56 patients were included and divided into two groups: 28 patients with GDM and 28 control patients without GDM. The 56 patients included in the study represented all consecutive cases scheduled for elective cesarean section during the specified period who met the inclusion criteria. All eligible patients who presented during this timeframe were enrolled in the study, and no additional randomization method was applied.

### Ethics approval and Informed consent:

The study was conducted after obtaining approval from the local ethics committee of Giresun University Faculty of Medicine (date: April 3, 2023, No: E-535593568-929212619451).All patients provided written informed consent after receiving a detailed explanation of the study procedure.

### Study population:

GDM was diagnosed based on the criteria set by the American Diabetes Association. For healthy individuals, fasting plasma glucose levels should be less than 92 mg/dL, and following a 75-gram glucose tolerance test, glucose levels should remain below 180 mg/dL at one hour and below 153 mg/dL at two hours. Values above these thresholds were considered to be indicative of GDM. Patients with a body mass index (BMI) between 25-35, aged 18 to 40 years, and classified as ASA1(American Society of Anesthesiologists physical status classification) were included in the study. Patients with comorbidities, contraindications to spinal anesthesia (such as infection at the injection site or clotting disorders), or undergoing emergency cesarean sections were excluded.

### Anesthesia procedure:

All patients received standard monitoring (noninvasive blood pressure, electrocardiogram and peripheral oxygen saturation) on arrival in the operating theatre. Spinal anaesthesia was administered by an anaesthesiologist using the same dose of local anaesthetic for all patients. Specifically, 12 mg of 0.5% hyperbaric bupivacaine (Marcaine®, AstraZeneca) was administered intrathecally. In the sitting position, a 25-gauge Quincke spinal needle was inserted into the L4-5 space.

### Sensory and Motor block assessment:

The level of sensory block was assessed using a pinprick test, and motor block was evaluated using the Modified Bromage(MB)^10^ scale:

• Score 0: No paralysis, full flexion of knee and foot.
• Score 1: Only the ability to move the knees and feet cannot lift the legs.
• Score 2: Unable to flex knees, able to move feet.
• Score 3: Complete paralysis, unable to move ankle or toes.

### Monitoring and Data collection:

Heart rate and blood pressure were recorded at baseline and 3, 5, 10, 15, 30 and 45 minutes after spinal anesthesia administration. The time to reach the T6 sensory block, time to achieve each score on the MB scale, highest sensory block level, and any anesthetic complications were recorded by a different anesthesiologist.

### Postoperative recovery and analgesia:

At the end of the surgery, the patients were transferred to the recovery room. Patients with sensory block levels below T6 and a Modified Aldrete score greater than eight were transferred to the surgical ward. The duration of the sensory block(from local anesthetic injection to the return of sensation at the S2 dermatome), duration of the motor block(from local anesthetic injection to full motor recovery), and time to the first postoperative analgesic requirement were recorded. Postoperative recovery and analgesia assessments were performed by an experienced anesthesiologist who was not directly involved in the study, following a standardized protocol. This approach was chosen to minimize observer bias.

### Statistical analysis:

All statistical analyses were performed using SPSS version 27.0. Quantitative variables are presented as mean, standard deviation, median, minimum, and maximum values, while qualitative variables are expressed as frequencies and percentages. The Shapiro-Wilk test and Box Plot graphs were used to assess the normality of the data distribution.

For normally distributed quantitative data, comparisons between two groups were made using Student’s t-test. For non-normally distributed data, the Mann-Whitney U test was applied. Within-group comparisons over time were conducted using the Repeated Measures test with Bonferroni correction for normally distributed data and the Friedman test with Bonferroni-Dunn correction for non-normally distributed data. Categorical variables were compared using Fisher’s exact test or Fisher-Freeman-Halton test. The results were considered statistically significant at a 95% confidence interval, with a p-value of less than 0.05.

## RESULTS

There were no statistically significant differences between the Non-GDM and GDM groups in terms of age, height, weight, BMI, or ASA (Table-I). When evaluating heart rate values based on diabetes mellitus (DM) status, it was evident that the 10th-minute heart rate of GDM patients was significantly higher than that of non-GDM patients. This difference was observed both in absolute terms(p=0.010) and in comparison with the baseline heart rate (p=0.004, p<0.01) (Table-II).

**Table-I:** Comparison Between Non-GDM and GDM Groups.

Characteristic	Non-GDM (n=28)	GDM (n=28)	p-value
Age (years)	Mean ± SD: 30.89 ± 5.17	Mean ± SD: 32.43 ± 4.60	0.246 ^a^
	Median (Min-Max): 30 (23-40)	Median (Min-Max): 33 (22 41)	
Weight (kg)	Mean ± SD: 85.86 ± 10.87	Mean ± SD: 82.86 ± 14.35	0.382 ^a^
	Median (Min-Max): 84 (66-106)	Median (Min-Max): 78.5 (62-117)	
Height (cm)	Mean ± SD: 163.00 ± 3.23	Mean ± SD: 161.54 ± 6.43	0.239 ^a^
	Median (Min-Max): 162 (150-170)	Median (Min-Max): 161 (140-170)	
BMI (kg/m²)	Mean ± SD: 32.39 ± 4.37 Median (Min-Max): 32 (25-40)	Mean ± SD: 32.96 ± 6.06 Median (Min-Max): 31 (24-50)	0.236 ^b^
Need for İnsulin	0(0%)	20(71%)	
ASA Classification	ASA I: 0 (0.0%)	ASA I: 3 (10.7%)	0.687 ^a^
	ASA II: 28 (100.0%)	ASA II: 25 (89.3%)	

GDM: gestational diabetes mellitus, Mean ± SD: Mean value and standard deviation, ASA: American Society of Anesthesiologists physical status classification, BMI: Body mass index.

a Student-t Test,

b FisherExact Test, **p<0,01.

Mean arterial pressure(MAP) values showed no significant differences between non-GDM and GDM patients at baseline and up to the 15th minute; however, GDM patients had significantly higher MAP values at the 30th minute (p=0.032) and 45th minute (p=0.001). Both groups experienced significant decreases in MAP from baseline over time, with notable drops at various time points, including the 30th and 45th minute, although the changes between groups were not significantly different (Table-II). Statistical significance(p<0.05) was found for Sensory Block Duration and Motor Block Duration, indicating a significant difference between the GDM and non-GDM groups for these variables (Table-III).

**Table-II:** Comparison of Heart Rate and Mean Arterial Pressure (MAP) Values According to Diabetes Mellitus (DM).

Heart Rate (HR)	Non-GDM (n=28)	GDM (n=28)	^a^p-value	MAP	Non-GDM (n=28)	GDM (n=28)	^a^p-value
Baseline	Mean±SD: 107.14 ± 12.06	Mean±SD: 104.57 ± 14.58	0.475	Baseline	Mean ± SD: 108.11 ± 12.57 Median(Min-Max):105.5(85-135)	Mean ± SD: 112.68 ± 15.68 Median(Min-Max): 110.5 (87-165)	^a^0.234
3rd minute	Mean±SD: 105.46 ± 22.61	Mean±SD: 102.57 ± 21.89	0.629	3rd min.	Mean ± SD: 86.54 ± 16.50 Median (Min-Max): 87 (60-119)	Mean ± SD: 88.18 ± 22.51 Median (Min-Max): 90.5 (47-145)	0.757
5th minute	Mean±SD: 98.86 ± 26.66	Mean±SD: 92.93 ± 23.68	0.383	5th min.	Mean ± SD: 78.50 ± 16.60 Median(Min-Max):76.5(55-117)	Mean ± SD: 81.54 ± 23.05 Median(Min-Max):81.5 (45-128)	0.574
10th minute	Mean±SD: 84.79 ± 15.64	Mean±SD: 97.29 ± 19.05	0.010*	10th min.	Mean ± SD: 83.21 ± 13.20 Median (Min-Max): 84 (60-113)	Mean ± SD: 84.25 ± 11.48 Median (Min-Max): 84.5 (64-114)	0.755
15th minute	Mean±SD: 90.25 ± 13.54	Mean±SD: 93.18 ± 17.43	0.486	15th min.	Mean ± SD: 80.57 ± 14.54 Median(Min-Max):76.5(50-114)	Mean ± SD: 81.07 ± 12.25 Median (Min-Max): 82.5 (59-105)	0.890
30th minute	Mean±SD: 91.18 ± 13.75	Mean±SD: 87.57 ± 16.14	0.372	30th min.	Mean ± SD: 77.68 ± 9.83 Median (Min-Max): 76.5 (63-97)	Mean ± SD: 83.50 ± 9.96 Median (Min-Max): 82 (65-105)	0.032*
45th minute	Mean±SD: 90.54 ± 13.23	Mean±SD: 90.39 ± 9.65	0.963	45th min.	Mean ± SD: 78.86 ± 8.70 Median (Min-Max): 77 (65-99)	Mean ± SD: 87.57 ± 10.65 Median (Min-Max):85(72-119)	0.001**

GDM – Gestational Diabetes Mellitus, Non-GDM – Non-Gestational Diabetes Mellitus (control group), MAP: Mean Arterial Pressure.

a Mann-Whitney-U Test,

** p<0,01,

* p<0,05.

**Table-III:** Comparison According to Diabetes Mellitus (DM) Characteristic.

Characteristic	GDM (n=28)	Non-GDM (n=28)	p-value
T6 Arrival Time (seconds)	150.0 (90.0 - 250.0)	170.0 (80.0 - 410.0)	^d^0.64
Highest Thoracic Level Reached	4 (2-5)	4 (2-5)	^d^0.37
Bromage Score 1 Arrival Time	85.0 ± 34.6	95.3 ± 30.6	^a^0.37
Bromage Score 2 Arrival Time	150.4 ± 57.6	162.5 ± 62.2	^a^0.53
Bromage Score 3 Arrival Time	258.57 ± 92.89	285.96 ± 98.85	^a^0.290
***Need for Ephedrine***			
No	12 (42.9%)	15 (53.6%)	
Single dose	13 (46.4%)	12 (42.9%)	^f^0.607
Two doses	3 (10.7%)	1 (3.6%)	
***Need for Atropine***			
No	28 (100.0%)	25 (89.3%)	^f^0.236
Single Dose	0 (0.0%)	3 (10.7%)	
***Need for Zofer***			
No	20 (71.4%)	17 (60.7%)	
Single Dose	4 (14.3%)	10 (35.7%)	^f^0.105
Two doses	0 (0%)	1 (3.6%)	
Sensory Block Duration (minutes)	155.4 ± 30.1	138.2 ± 25.6	^a^0.005**
Motor Block Duration (minutes)	178.0 ± 42.5	152.7 ± 30.0	^a^0.002**

GDM – Gestational Diabetes Mellitus, Non-GDM – Non-Gestational Diabetes Mellitus (control group)

a Student-t Test,

d Mann-Whitney-U Test,

f Fisher Freeman Halton Test,

** p<0,01,

*p<0,05.

## DISCUSSION

Our findings indicate that patients with GDM exhibit significantly prolonged sensory and motor block durations during regional anesthesia compared with non-GDM patients. Additionally, GDM patients showed delayed cardiovascular responses, including higher heart rates at the 10th minute post-anesthesia, as well as increased MAP at the 30th and 45th minutes.

These findings are in line with existing literature on anesthetic management in patients with diabetes.^10^-^12^ This is consistent with our finding of extended sensory and motor block durations in GDM patients, highlighting the need for tailored anesthesia protocols for this patient population.^13^ Moreover, the delayed cardiovascular responses observed in our study are consistent with the reports of autonomic dysfunction in patients with diabetes. GDM patients exhibit significantly higher heart rates and MAP values during anesthesia, suggesting that autonomic neuropathy may contribute to altered cardiovascular regulation.^9^,^14^-^17^

Diabetic peripheral neuropathy (DPN) is a common complication of diabetes, affecting approximately 50% of patients with diabetes. Since all local anesthetic agents have some degree of neurotoxicity, there is concern that already damaged diabetic nerves may be more susceptible to additional harm. This increased vulnerability is referred to as the “double-crush” effect, where pre-existing metabolic damage to diabetic neuropathic nerves heightens the risk of anesthetic toxicity.^18^,^19^ However, the extended duration of nerve blocks in patients with diabetic neuropathy reduces the need for vasoconstrictive adjuvants. Therefore, routine use of local vasoconstrictors in diabetic patients is not generally recommended.^14^,^20^,^21^

Diabetic nerves exhibit increased sensitivity to local anesthetics, resulting in higher success rates and longer-lasting nerve blocks, especially in patients with diabetic neuropathy. Studies by Kroin and Lirk have shown that diabetic neuropathic animals experience extended block durations.^22^,^23^ Studies have demonstrated that peripheral and spinal nerve blocks last longer in patients with diabetes than in healthy individuals. This extended duration was attributed to both pharmacodynamic and pharmacokinetic factors. Pharmacokinetically, reduced blood flow and perfusion in diabetic neuropathic nerves causes anesthetics to remain effective for longer periods. This leads to prolonged and more intense blocks, which can increase the risk of complications, such as pressure ulcers and delayed mobility, especially when high concentrations of anesthetics are used in extended blocks.^6^,^9^,^15^,^22^-^24^

Few studies have examined the impact of sex differences on peripheral nerve block despite significant baseline differences between patients with and without diabetes. Both clinical and animal studies suggest that non-pregnant women are more sensitive to intrathecal bupivacaine during spinal anesthesia than men, and this sensitivity is heightened during pregnancy due to elevated progesterone levels. This increased nerve sensitivity during pregnancy also extends to peripheral nerve blocks. Tang et al. found that female patients with diabetes experienced significantly longer sensory and motor nerve blocks than female patients without diabetes; however, this difference was not observed in male patients. However, the mechanism underlying this discrepancy remains unclear.^24^

The reduced nerve conduction velocity and microvascular dysfunction that occur in diabetes can impair blood flow to the nerves, prolonging anesthetic block effects.^6^ This phenomenon was observed in our study, further supporting the notion that anesthetic management in patients with GDM requires careful adjustment to avoid excessively prolonged recovery times. Our findings emphasize the importance of individualized anesthetic management in GDM patients. Prolonged sensory and motor block durations along with delayed cardiovascular responses necessitate careful planning and monitoring to optimize patient outcomes and minimize risks during surgery. Preoperative assessments should include evaluation of neuropathy severity and other comorbidities, and anesthesia protocols should be adjusted accordingly. The additional use of local vasoconstrictors in patients with diabetes is not routinely recommended.

Further research is needed to elucidate the molecular mechanisms underlying prolonged nerve block duration in patients with diabetes. Additionally, future studies should investigate potential interventions that could mitigate the effects of neuropathy on spinal anesthesia outcomes. The role of sex and hormonal differences in anesthetic responses, particularly in women with GDM, should also be explored to improve tailored anesthetic care.

### Strengths of the study:

This study has several notable strengths. First, it focused on a highly relevant and increasingly common clinical condition—GDM—using a standardized spinal anesthesia protocol, which minimized variability between patients. Second, the prospective and controlled design enhanced the reliability of the findings by reducing recall and selection bias. Third, consecutive patient enrollment ensured that the study population closely reflected real-world clinical practice. Finally, detailed intraoperative hemodynamic monitoring allowed for precise comparison of cardiovascular responses between groups. These strengths increase the clinical relevance and generalizability of the findings, despite the limitations discussed.

### Limitations:

The generalizability of this study has several limitations. The limited sample size of 56 individuals and the use of a single center makes it difficult to confidently generalize the results to a larger population. Utilizing a single healthcare institution for the study further limits the range of patients included; a multicenter strategy would yield more representative data. Finally, the results are not applicable to other forms of anesthesia or surgical settings because of the focus of the study on spinal anesthesia during cesarean sections.

## CONCLUSION

GDM patients exhibited prolonged sensory and motor block durations, suggesting altered nerve function or drug metabolism under anesthesia. Moreover, the delayed cardiovascular responses observed, including elevated heart rates and increased MAP at various time points, indicate a heightened cardiovascular sensitivity in GDM patients. These findings highlight the necessity of tailoring anesthesia protocols specifically for patients with GDM, with close perioperative monitoring of cardiovascular function. Larger multicenter studies are needed to confirm these results and to investigate whether alternative anesthesia techniques may improve outcomes in this population.

### Acknowledge:

AI was used in the preparation of this manuscript to improve English language and Grammar.
